# Between rights and reality: patient perceptions of social inclusion in a forensic psychiatric clinic implementing safewards

**DOI:** 10.3389/fpsyt.2025.1688564

**Published:** 2025-11-19

**Authors:** Veikko Pelto-Piri, Lars Kjellin, Linn Kronman, Jenny Wetterling, Anna Björkdahl

**Affiliations:** 1 Faculty of Medicine and Health, University Health Care Research Center, Örebro University, Örebro, Sweden; 2 Faculty of Medicine and Health, School of Medical Sciences, Örebro University, Örebro, Sweden; 3 Department of Clinical Neuroscience, Centre for Psychiatry Research, Karolinska Institutet & Stockholm Health Care Services, Stockholm, Sweden

**Keywords:** safewards, forensic psychiatry, social inclusion, human rights, vulnerable populations, coercive measures, violence prevention, implementation

## Abstract

**Background:**

Upholding human rights, including promoting patients’ social inclusion, is essential in forensic psychiatric services to ensure socially sustainable development. Understanding how social inclusion it is perceived by patients is therefore important. Evidence suggests that the Safewards model can enhance social inclusion in inpatient settings. The aim of this study was to explore patients’ perceptions of social inclusion in a forensic psychiatric clinic that implemented the Safewards model. To provide context, a secondary aim was to describe which Safewards interventions were implemented and how patients perceived them.

**Methods:**

Eight male patients from a forensic psychiatric clinic in Sweden were individually interviewed, focusing on Safewards and opportunities for social inclusion. Thematic content analysis was used to deductively code the data under the three core values of a social inclusion model: participation in decision-making, reciprocal relationships, and social justice. Themes for each value were thereafter developed inductively. For the secondary aim, a concise qualitative description was used to create an overview of patients’ perceptions of Safewards.

**Results:**

Patients were aware of the Safewards interventions and were generally positive about them. Nine themes emerged: powerlessness when meeting the doctor; limited experience of involvement in daily decisions; desire to become involved in quality improvement efforts (participation in decision-making); staff members’ availability and positive attitude; fellow patients exhibit care and consideration; withdrawal and avoidance of social interaction (reciprocal relationships); boredom and low mood within the ward; desire for dignified, equal, and safe care; and perception of unjustified confinement (social justice).

**Conclusion:**

The findings suggest that while Safewards may support more compassionate staff–patient relationships, it does not in itself ensure participatory or rights-based care. Achieving higher levels of social inclusion in forensic psychiatric services requires broader cultural and structural reforms beyond the implementation of individual models.

## 1 Introduction

“Social inclusion is the process by which efforts are made to ensure equal opportunities - that everyone, regardless of their background, can achieve their full potential in life. Such efforts include policies and actions that promote equal access to (public) services as well as enable citizen’s participation in the decision-making processes that affect their lives.” – Social inclusion as defined by Division for Social Policy and Development, United Nations (1).

The United Nations (UN) Convention on the Rights of Persons with Disabilities (CRPD) aims to promote the social inclusion of persons with disabilities (2). Member states that have ratified the convention should take legislative and policy measures to eliminate discrimination against this group. The presence of a disability should not justify deprivation of liberty or the use of coercive measures. Sweden has faced extensive criticism for psychiatric practices that conflict with the principles of the CRPD (3, 4). The UN Committee monitoring the convention has repeatedly criticized Swedish psychiatry for overuse of coercive measures, inadequate documentation and a narrow biomedical perspective, urging the state to improve care and transparency (3, 5). Similar concerns have been raised by Sweden’s national monitoring body under the UN Convention against Torture and Other Cruel, Inhuman or Degrading Treatment or Punishment (CAT), which has condemned the frequent use of coercive practices in forensic psychiatric services (6). Patients in Swedish forensic psychiatric services often present with multiple and complex needs, including comorbid psychiatric disorders, substance use, cognitive impairments, and various social vulnerabilities (e.g., immigrant or refugee background, low educational attainment) (7–9), which may create barriers to participation and communication. The combination of these factors, together with paternalistic and custodial institution culture, make implementing human rights conventions particularly demanding.

The UN Sustainable Development Goal No. 16 aims to promote the development of effective, accountable and transparent institutions to ensure responsive, inclusive, participatory and representative decision-making at all levels (Target 16:6) (10). Social inclusion is a fundamental foundation for achieving socially sustainable development, as it operationalizes human rights values, such as participation, dignity, equality, and non-discrimination, within institutions (11–13). From this perspective, social inclusion represents both a process and an outcome that enables individuals, particularly the most vulnerable, to exercise their rights and participate fully in society (11, 14). Such efforts provide individuals with opportunities to actively participate and make their voices heard (10, 15, 16). At the beginning of the 21st. century, a policy of social inclusion was introduced in many organizations working with disabled persons, creating a need to measure and evaluate it (12, 13, 17). However, the instruments developed were criticized for their narrow definitions of social inclusion, their tendency to disregard the inherent power imbalance between users and service providers (16, 17) and failure to address social inclusion as a context-dependent set of values rather than a narrowly defined empirical concept (11, 18). In psychiatric services, understanding of social inclusion as a set of core values is appropriate, as the World Psychiatric Association and The World Association of Social Psychiatry have adopted several declarations that align with human rights and incorporated social inclusion in their psychiatric ethical framework (19–21).

In this paper, we define social inclusion in inpatient psychiatric services as consisting of three core values: Participation in decision-making, reciprocal relationships and social justice (**Table 1**) (21, 22), a definition that aligns with earlier and contemporary literature (17).

**Table 1:** Core values of social inclusion in institutional psychiatric settings, modified from Pelto-Piri & Kjellin, 2021 (21) and Pelto-Piri & Wetterling 2024 (22).

Core values	Patient seen as a	Organizational perspective	Goal of care
Participation in decision-making	Patient	Patient rights	Informed patients influencing the care provided by staff
Reciprocal relationships	Person	Person-centered, recovery-oriented and other strength-based approaches	The knowledge and experiences of both patient and staff are used to create recovery strategies
Social justice	Citizen	Accountable and transparent organization	Access to -friendly and non-discriminatory services

The first core value in the model (**Table 1**) concerns patients’ rights to participate in their care and treatment, a principle that emerged in medical ethics after World War II as a reaction to the paternalistic tradition in medicine (20). According to the World Medical Association’s Code of Medical Ethics, physicians are obliged to inform patients at every stage of the care process and to offer care that aligns with the patient’s best interests, to which the patient has the right to consent or refuse (23, 24). These criteria represent the minimum standard for participation. Today, patients may also be offered multiple options for their care and the possibility of seeking a second opinion. Shared decision-making can be considered part of participation (25). However, in this model, it is categorized under the second core value, reciprocal relationships, which is emphasized in person-centered, recovery-oriented, and other strength-based approaches. It highlights that human relationships are essential for survival and health (26) as well as each individual’s right to mutual relationships with others (27, 28). The values underlying these approaches are closely interrelated and can be viewed together, following the WHO’s description of a collaborative model of health and social care in which the individual’s unique goals, strengths, and preferences are central to care planning and decision-making. The core value of reciprocal relationships highlights the creation of trusting and mutual relationships based on respect, empathy, and understanding. It seeks to promote dignity, autonomy, and meaning in life rather than focusing solely on symptom reduction. From this perspective, the individual is recognized not merely as a recipient of care but as an active partner whose experiential knowledge complements professional expertise (25, 29). The third core value, social justice, concerns recognizing patients as citizens and right-holders in relation to the psychiatric services, which are duty-bearers (24, 30). Social justice partly refers to the fair distribution of resources, opportunities and privileges within society (31) and is especially important when working with vulnerable populations, such as forensic psychiatric patients (32). It is also a part of psychiatric ethics, implying that staff should engage in promoting patients’ civil rights (27, 28, 31, 32). According to the European Standard on Patient Involvement in Health Care (24), this responsibility extends beyond individual staff members to the organizational level, requiring healthcare institutions to provide the structural and cultural conditions that enable professionals to practice person-centered and participatory care. Furthermore, social justice also implies that patients and their organizations should be encouraged to participate in the co-creation and co-production of psychiatric services (22). The recovery perspective emerged from the social justice movement. However, it has been suggested that it has since been co-opted and mainstreamed by professionals and policymakers, who have subsequently downplayed the original emphasis on social justice (33, 34).

It has been suggested in the literature that forensic psychiatric services should introduce a value basis with a stronger focus on human rights, as this would provide opportunities to adopt a strength and recovery-oriented practice (35). One way to support such a shift may be through the implementation of the Safewards model to reduce coercion and improve ward climate, an approach that this study sought to explore. The Safewards model was originally developed to prevent conflict and containment in acute psychiatric wards and consists of ten interventions (36) but is now also being used in many forensic psychiatric services (37). Frequently the primary reason for implementing Safewards in forensic psychiatric services is not reduction of coercive measures, which are relatively few compared with acute psychiatry. Instead, Safewards addresses important challenges in forensic psychiatric care, such as creating a sense of community and hope, communication strategies and early support for patients in need of care (**Table 2**) (36, 38). Safewards could thus facilitate a shift from the traditional paternalistic and custodial culture towards a more inclusive care culture (39). When Safewards was implemented in forensic psychiatric services, a need for adaptations was observed and research has been conducted on developing such adaptations (39, 40).

**Table 2:** The ten Safewards’ interventions for reducing conflict, from Fletcher et al., p. 3 (38).

Intervention	Description	Purpose
Discharge Messages	Before discharge, patients leave messages of hope for other patients on a message board in the unit	Strengthens the patient community, generates hope
Know Each Other	Patients and staff share some personal interests and ideas with each other, which are displayed in common areas of the unit	Builds rapport, connection and a sense of common humanity
Clear Mutual Expectations	Patients and staff work together to create mutually agreed aspirations that apply to both groups equally	Counters some power imbalances, creates a stronger sense of shared community
Mutual Help Meetings	Patients offer and receive mutual help and support through a daily, shared meeting	Strengthens the patient community, opportunity to give and receive help
Positive Words	At handover time, staff members say something positive about each patient, using psychological explanations to describe challenging actions	Increases positive appreciation and provides helpful information for colleagues in their work with patients
Soft Words	Staff take great care with their tone of voice and use of collaborative language. Staff reduce the limits faced by patients, create flexible options and exhibit respect if limit setting is unavoidable	Reduces a common flashpoint, builds respect, choice and dignity
Bad News Mitigation	Staff understand, proactively plan for and mitigate the effects of bad news received by patients	Reduces impact of common flashpoints, offers extra support
Calm Down Methods	Staff support patients to draw on their strengths and promote coping skills before prescribing PRN medication or containment	Strengthens patient confidence and skills to cope with distress
Talk Down	De-escalation process focuses on clarifying issues and finding solutions together. Staff maintain self-control, respect and empathy	Increases respect, collaboration and mutually positive outcomes
Reassurance	Staff touch base with every patient after every conflict on the unit and debrief as required	Reduces common flashpoints, increases patients’ sense of safety and security

To ensure the social sustainability of forensic psychiatric services, there is a growing expectation for the field, internationally and in Sweden, to adopt more person-centered, recovery-oriented and other strength-based approaches (41, 42). This shift can be achieved by promoting an institutional culture that upholds human rights and enhances patients’ social inclusion (43). To advance forensic psychiatric services in this way we need a deeper understanding of how social inclusion is perceived by patients. Although there are some challenges involved in adapting and implementing the Safewards model in forensic psychiatric services, evidence suggests that it can enhance social inclusion, particularly in terms of how patients perceive their situation (37, 44). The aim of this study was to explore patients’ perceptions of social inclusion in a forensic psychiatric clinic that implemented the Safewards model. To provide context, a secondary aim was to describe which Safewards interventions were implemented and how patients perceived them.

## 2 Materials and methods

### 2.1 Design

This interview study employed qualitative content analysis and qualitative description to examine data from semi-structured interviews (45–47). These methods were chosen to provide a comprehensive account of patients’ narratives within the framework of social inclusion, focusing on their experiences of living in a forensic psychiatric clinic implementing the Safewards model. To ensure methodological rigor, the Consolidated Criteria for Reporting Qualitative Research (COREQ) checklist was applied (48).

### 2.2 Settings and sample

The study was conducted in a clinic that primarily provides care for individuals sentenced within the catchment area of a Swedish county with approximately 300, 000 inhabitants. According to national classifications, it is considered an intermediate region, with an urban center surrounded by smaller municipalities and rural communities. The clinic consisted of four wards that predominantly, but not exclusively, treated male patients. It had 27 beds in three low-security wards and seven in a medium-security ward, which also functioned as an admission ward. One of the low-security wards served as a discharge ward. This arrangement meant that patients were transferred between wards during their care. The clinic started to implement Safewards in 2020. The wards had implemented three to five interventions. While all wards included Talk Down in their training for managing aggression and violence, only two wards had integrated it as part of their work with Safewards. One of the wards worked especially hard on Calm Down Methods. They had furnished a small, doorless room called the calm room in a homely style with two armchairs. This room was appreciated by patients and primarily intended for use in the Calm Down Methods intervention but was also used for conversations. All wards conducted patient meetings. However, only one had implemented the Mutual Help Meeting intervention.

The interviews were conducted at the turn of the year 2022/23. We applied three exclusion criteria: Patients who were (1) unable to provide informed consent, (2) acutely ill or exhibited violent behavior, making an interview inappropriate and (3) unable to participate in an interview due to insufficient proficiency in Swedish. A nurse provided a list of nine patients who met the inclusion criteria. All eligible patients were interviewed, except for one individual who was on leave during the scheduled visits. At the time of recruitment, the clinic had 34 patients, including three women who were excluded based on the agreed criteria. Eight male patients were interviewed, their ages ranging from 21 to 63 years, with a mean age of 40 years.

### 2.3 Interview guide and procedure

A semi-structured interview guide (see **Supplementary File 1**) was used, with questions about social inclusion at the ward, the implemented Safewards interventions and Safewards’ contribution to a safe ward environment. The interviewers did not have any relationship established with the patients prior the study commencement. The patients were informed about the study both verbally and in writing by the nurse in charge, who also asked about their interest in participating. The interviewers later repeated the study information, and all participants gave their written consent to participate. Individual interviews were conducted in a room at the ward. Prior to the interviews, participants were informed that the interviews would be conducted by a research assistant (see Acknowledgements) and a researcher (VP, see Authors’ Contributions), both of whom had personal experience as patients in psychiatric inpatient care. The purpose of this disclosure was to be transparent and honest with the participants, possibly influencing their perception of the interviewers as credible and trustworthy and, consequently, what experiences they chose to share (49). The interviews, which lasted from 23 to 50 minutes (mean 35 minutes), were recorded and transcribed verbatim.

### 2.4 Analysis and interpretation

To address the supplementary aim and provide an overview of patients’ experiences of Safewards, nine Safewards interventions were used as categories to generate a concise qualitative description (46, 47). The *Positive Words* intervention was excluded, as it relates to staff handovers, during which patients are not present.

To address the main aim, a qualitative content analysis was employed (45). The three theoretical core values of the social inclusion model served as an analysis matrix for deductively categorizing the interview data. Following this initial categorization, an inductive analysis was conducted within each category to identify emerging themes. Initially, the four researchers (VP-P, LiK, LaK, JW) were each assigned one interview to read thoroughly, marking relevant sentences and paragraphs, which were then categorized under the social inclusion core values. Any ambiguities or uncertainties regarding coding were discussed and resolved through consensus. The interviews were then divided among LiK, LaK and JW, who proceeded to code the entire dataset in Excel after which the codes were checked by another of the three researchers in this group. At our second meeting, we addressed problems involving interpretation and overlapping coding within the social inclusion categories and agreed on a consistent approach. At the third meeting, we reviewed the analyzed material, identifying recurring patterns and key insights. We selected significant quotations that exemplified social inclusion and organized them into themes. As the last step, during our concluding meeting and final interpretation, all authors reviewed the potential themes and collectively selected those that best represented the interview content. VP and AB, who were involved in the implementation of Safewards in their respective regions, did not conduct primary analyses, such as coding of interviews, but contributed to the discussions during the entire analysis process.

### 2.5 Reflexive practice and positionality

The research team, consisting of senior researchers, experts by experience (from acute and forensic psychiatric inpatient services, but not from the included clinic) and a medical master student, engaged in ongoing reflexive practice throughout the study to address their positionality and potential influence on the research process. Three individuals, one interviewer and two authors, had lived experience of mental health issues and psychiatric inpatient care. This diversity enriched the research process but also necessitated critical reflection. Throughout the research process, the team held discussions about their positionalities and preconceptions related to the research topic, ensuring an awareness of the risk of bias as well as enhancing the rigor and trustworthiness of the study (50).

## 3 Results

Before presenting the main findings, a brief description is provided of the Safewards interventions implemented in the four wards and participants’ perceptions of them. Our findings suggest that most participants were aware of the Safewards interventions and generally viewed them positively, see **Table 3**.

**Table 3:** Participant perceptions of Safewards in the four wards of the clinic.

Interventions	Used in ward*	Patients’ perceptions of the interventions in Safewards
Discharge Messages	4	Those who were aware of the intervention considered it positive, both challenging and encouraging hope.
Know Each Other	2, 4	Experienced positively, good for those who are not socially skilled. Additionally, patients participated in the intervention.
Clear Mutual Expectations	1, 2, 4	Contributes to safety at the ward. Knowing what rules apply is good, but the rules are not developed together with the patients.
Mutual Help Meeting	3	There were meetings, but these were not described as Mutual help meetings. Patients had limited participation in decision-making and the meetings were not described as supportive. Many patients chose not to participate.
Soft Words	2, 3, 4	Staff members’ communication with patients was most often good, but feelings of an “us and them” atmosphere were also described.
Bad News Mitigation	1	Staff members’ presentation of bad news was sensitive with a calming attitude, although they sometimes communicated bad news in the presence of other patients, which was not appreciated.
Calm Down Methods	1, 4	Appreciated by most patients, but they wished staff would offer such methods more often instead of patients having to ask for them.
Talk Down	1, 3	Staff members used calm down methods to defuse situations as well as being attentive and responsive.
Reassurance	1, 3	Examples of staff not reassuring patients after an incident, as well as good examples of when they did provide reassurance.

*"Used in ward" denotes the ward(s) where each respective intervention was implemented at the time of the interviews.

To address the main aim, we created nine themes in the analysis grounded in the core values of social inclusion: participation in decision-making, reciprocal relationships, and social justice (**Table 4**).

**Table 4:** Overview of the thematic framework.

Core values	Themes
Participation in decision making	Powerlessness when meeting the doctor Limited experience of involvement in daily decisions Desire to become involved in quality improvement efforts
Reciprocal relationships	Staff members’ availability and positive attitude Fellow patients exhibit care and consideration Withdrawal and avoidance of social interaction
Social justice	Boredom and low mood within the ward Desire for dignified, equal and safe care Perception of unjustified confinement

### 3.1 Participation in decision-making

#### 3.1.1 Powerlessness when meeting the doctor

Participants stated that the doctor has a plan regarding their care that cannot be influenced by the patient’s own thoughts or opinions. The feeling was that the doctor has overwhelming power regarding care content and decision-making and does not take patients’ opinions into account. The participants were frustrated about the lack of participation in their own care process, which increased when they felt that their voices were not heard or taken seriously.

“You know, there’s really nothing you can have a say in. The doctors have a plan, and that’s the plan that sticks: you have to get treatment, find a job, get a flat, then move on to outpatient care—and once you’re through that, you’re discharged.” – Participant 7.

The participants expressed that they had few meetings with the doctor with too much time elapsing between meetings and that their mental health status could change significantly in the meantime. Although they did not have many meetings with the doctor, they described the doctor as having full insight into their situation. A sense of being under constant observation intensified feelings of powerlessness.

Interviewer: Have you ever noticed, if someone doesn’t follow the rules, what happens then?

Participant 3: Yes, they write it down in, um, some book or make a note.

Interviewer: But what do the staff do then? For that patient, besides making a note or something?

Participant 3: They tell the doctor. So, it’s up to the doctor then. I’ve experienced that.

#### 3.1.2 Limited experience of involvement in daily decisions

The participants expressed that their ability to make everyday decisions was limited to a weekly meeting, where they were allowed to choose activities for the weekend, which toppings to have on their sandwiches and what type of bread they want with their coffee. Although these choices were relatively minor, they were nonetheless perceived as meaningful and important aspects of everyday life.

“Yeah, I mean, it’s the little things, really—like choosing what spread you want [for breakfast] or stuff like that. It’s part of feeling involved, you know? You might not get a say in the big, important stuff, but choosing your spread can feel like a big deal when you’re having breakfast. Like, getting your egg instead of nothing.” – Participant 5.

These meetings appeared to be the only occasion when patients had some sort of influence on the ward. Some participants described a feeling of powerlessness when it comes to important aspects of their own care and daily life at the clinic.

#### 3.1.3 Desire to become involved in quality improvement efforts

Participants made suggestions about how to improve everyday life and the atmosphere in the ward to increase the feeling of normality and make it more homely. There were requests and ideas for more meaningful activities, such as cooking together with other patients and staff. They wanted activities that could provide opportunities for socializing with others to promote the feeling of inclusion and belonging. For example, a participant expressed that the design of the Safewards Clear Mutual Expectations intervention was flawed. Although he believed the expectations were positive and contributed to a better atmosphere, it felt frustrating not to have been involved in the process of creating them.

“I mean, sure, it says good things, absolutely, but it’s not something we, the ones actually sitting in here now, have been part of creating together. That’s why it feels so off, you know, when I read ‘The mutual’. We’ve not been a part of it. I do understand that it’s meant to be there, like, it’s got a purpose—to guide how we’re supposed to behave towards each other and the staff and all that. And yeah, I understand that, I do, but still.” – Participant 1.

### 3.2 Reciprocal relationships

#### 3.2.1 Staff members’ availability and positive attitude

Staff members were generally described as positive and accessible, striving to create a supportive environment for the patients. Most of the staff worked actively to promote participation in activities. Participants appreciated the support and encouragement they received from the staff. They listened to patients with respect, which contributed to an atmosphere of understanding and empathy.

“Yes, but I have to admire the staff because when they notice that someone wants to talk and so on, they usually ask. So, you go aside and sit in the calm room, you know, so yes, they’re good at that.” – Participant 8.

An atmosphere of calm and well-being was created for patients through conversations and interactions, including playing cards and offering massage to reduce anxiety. Staff handled most situations in a calm, professional manner and treated patients in an individual way, creating a feeling of personal care and a stable, safe atmosphere on the ward. Many staff members were described as skilled in using the Talk Down intervention, calmly communicating with distressed or aggressive patients. Despite many positive descriptions of staff, some staff members were described as more stressed and less empathetic than others. Their work was more routine based, and they treated all patients in the same way, leading to a feeling of non-individual care. Participants described that this could reinforce their sense of illness rather than promoting their well-being.

“Yeah, but some are more stressed—only stressed when they’re here—while others stay calm and just take things easy, go about it methodologically. And then you’ve got the ones who are like, ‘What do we do now?’ You know, just running around. Like, go home instead! What are you even doing here? It just makes things stressful. Of course we feel that.” – Participant 1.

Participants described being influenced by each other’s moods. If someone was nervous or stressed, the rest of the patient group was negatively affected. In such situations it was especially crucial for staff to act in a stable and calm manner. If staff members were perceived as stressed or lacking empathy and not maintaining a consistent level of support, it could create anxiety and insecurity within the patient group.

#### 3.2.2 Fellow patients exhibit care and consideration

Participants generally expressed that fellow patients had an empathetic and considerate attitude, for example, they usually buy things for each other when out shopping.

“Yeah, but we share sweets and stuff like that. I went shopping the day before yesterday for a bloke who’s not allowed out—he chucked 60 kronor at me and said, ‘Get as much sweets as possible.’ So, I did. The atmosphere’s good and all that. But then, not everyone’s doing great either. Eh, but I reckon everyone wants the best for each other—no one’s putting anyone down or anything. So that’s a positive.” – Participant 4.

According to participants, there was mutual understanding and concern between patients. They were aware of each other’s needs and feelings and paid attention when other patients were feeling unwell. One participant thought that the presentations of persons in the Know Each Other intervention was good for those with less social skills, enabling them to feel more involved in the ward’s social community. There were also descriptions of patients who had a negative and complaining attitude, from whom participants chose to distance themselves from.

Interviewer: Another way, and yes, but this thing of showing consideration, seeing each other and being able to get comfort and feel hope both from other patients and staff, what is that like?

Participant 7: It depends on how you talk to them. You know, I only talk to my friends and so, my friends or those I hang out with. And they are very supportive. They say, ‘Come, you’ll be out soon, come to the flat, you will be fine,’ and so on. But there’s a whole lot of other rubbish with other patients where they sit and complain every day and make you think about being really locked up. They are the ones who need comfort the most, I think.

#### 3.2.3 Withdrawal and avoidance of social interaction

Participants reported that many fellow patients withdrew and avoided interaction with both staff and fellow patients. Although staff encouraged activities and social engagement, some patients preferred to remain in their rooms.

“No, I don’t know. They stay in their rooms. I’m more out in the day room during the day. Most people stay in their rooms.” – Participant 6.

They also described a lack of interesting activities and a generally low mood on the ward. At the same time, some participants actively sought social interaction and wished to engage with both staff and fellow patients. Furthermore, the extent to which participants felt safe with staff and other patients influenced their decision to either stay in their rooms or socialize with others.

Interviewer: Does it appear that staff members have a common view on how to work and how to treat patients, or does it differ between different staff members?

Participant 2: It differs. The ones I have the best contact with, um, are a bit more open with me. I talk more with them, we play cards, and then there are staff members I have maybe no contact with at all. I don’t even talk to them.

Interviewer: Do you act differently when those staff members are on duty.

Participant 2: Um, it depends. Some days, it’s only those staff members who are working and then I keep to myself. I’m in my room, mostly.

Interviewer: Okay.

Participant 2: And then I think that if something were to happen that day, I probably wouldn’t go to them.

### 3.3 Social justice

#### 3.3.1 Boredom and low mood within the ward

Participants described that the four wards were characterized by a general feeling of boredom, vulnerability and lack of activity. There were participants who expressed that most days were monotonous and uneventful, with only occasional days perceived as meaningful. Another participant described a boring daily routine where the days merged together without any variation.

“Yeah, not much happens. Just drink coffee all day, that’s probably the only thing you do. Coffee, smoke, take medicine, that’s probably all you do and go out sometimes.” – Participant 6

Participants also noted that their fellow patients tended to keep to themselves in their rooms, which added to the general feeling of boredom and isolation. The food was perceived as poor and the possibility of creating homely and comfortable environment was limited, as personal items such as rugs and blankets were not allowed. The staff said that it was not possible to have such items in the room due to the risk of fire. Participants were reminded by staff that hospital rules apply. For example, there was a rule in one of the wards that forbade patients from putting their feet on the coffee table, which one participant mentioned as being something you would normally do in a home environment. The participants suggested that more activities should be offered, such as eating or cooking food with more variety and from other cultures or having coffee outside the ward to break the monotony of everyday life.

“Sometimes we go to the clinic’s small cottage. I’ve never been there before, but I know they do it when it’s not as cold as it is now. And then … yes, sometimes you might be allowed to help in the kitchen and cook or something.” – Participant 3

#### 3.3.2 Desire for dignified, equal and safe care

The participants expressed that the feeling of safety in the ward was important, and they mostly experienced it as a safe place. Some participants perceived their stay at the ward as safer compared to their life outside the institution. The Clear Mutual Expectations contributed to this feeling of safety. Without a clear structure for what is allowed and what is prohibited, it could be difficult for patients to navigate and feel safe.

“Yes, but it’s that rules are followed and, and that everyone has the same value and so on. So, everyone is equal. No one should have more rights than anyone else, the same rule should apply to everyone.” – Participant 4

A participant described the uncertainty that arose when rules were not clearly defined. There were also experiences of uncertainty and anxiety, especially when faced with new incidents and patients. For example, a participant expressed being “on guard” all the time. The importance of being treated with dignity and respect was highlighted. At the same time there were experiences of exclusionary and non-empathetic meetings with certain staff members and fellow patients. For example, staff could choose not to engage in conversation with a patient who, according to one participant, they perceived as intimidating. Participants also emphasized the importance of everyone being treated equally, which was generally the case in the ward, although occasional incidents of favoritism or special treatment by staff were also reported.

“I think there are certain rules that only apply to certain people. Let’s say there’s a patient who has rings. Now, we’re not allowed to have rings here, but because this patient has outbursts, she can keep her rings. But me, who does nothing, I’m not allowed to have rings precisely because I do nothing. Eh, they don’t dare to take her rings precisely because she has outbursts like that.” – Participant 2.

#### 3.3.3 Perception of unjustified confinement

Participants expressed the unfairness of being locked up for many years, for example, for harassment or theft. They were aware of that a forensic psychiatric sentence in Sweden is not time-limited and typically results in a significantly longer period of care compared to a prison sentence.

Interviewer: Would you like to have more of a say?

Participant 6: No, I don’t know. I don’t think there’s any point. You just have to stay off drugs and keep calm in here, and eventually, I’ll be discharged. But I think it’s cramped in here. I’ve got … [a conviction for] harassment—even though I didn’t do it, for example. So I shouldn’t be here. … Sitting here, wrongly convicted, judged based on old records. Too many times.

Interviewer: But when you say that there’s no point in participation, how do you mean? Can you explain a bit more?

Participant 6: No, it’s just hopeless. I see this as a punishment and nothing else. Whether I’m guilty or not doesn’t matter, you get punished anyway. It’s like a punishment.

Several patients at the clinic were not allowed to be discharged from inpatient care, even though both they and the doctor considered that they were ready to leave. The reason for this was the lack of available housing and support.

“And the other patients, they liked me and all. And no, I don’t know, but it was a good time for me anyway. It was when I had come out of the psychosis and started to feel really good, you know. So, everything was going smoothly and I felt that soon I would probably be discharged. And I was. But now, now even though you feel healthy and well, it’s this thing about the flat, isn’t it? The doctors have said it themselves that only if I have a flat will I start outpatient care and be given leave so that it’s already sorted (sighs).” – Participant 8.

This situation was described as frustrating and a limitation for the patients. They felt that their opportunity to reintegrate into society is delayed by having to wait for suitable accommodation, leading to the risk of becoming institutionalized as well as limiting their opportunities to regain independent living and participation in society.

## 4 Discussion

This study highlights a tension between the ideals of human rights and the lived realities of patients in a forensic psychiatric clinic implementing Safewards. While Safewards aims to promote a more recovery-oriented and inclusive environment (51), our findings show that patients’ experiences of social inclusion remained limited. Safewards appears to have potential for fostering social inclusion, as its implementation in the clinic was generally well-received by the participants in our study. Participants described predominantly positive reciprocal relationships with staff members and fellow patients but the organization as such had not become more inclusive. Participants’ knowledge and experiences were seldom actively sought, and they reported limited opportunities to participate in decision-making in their care and daily lives. Regarding social justice, the ward environment was often considered dull, with limited activities and few forums for meaningful social interaction. Participants could feel that their time on the clinic resembled punishment rather than treatment, expressing frustration about prolonged care durations. This was especially true for those who could not be discharged due to the municipality’s inability to provide housing and support after their treatment had ended.

### 4.1 Participation in decision-making

The paternalistic tradition in health care generally, and especially in psychiatric services, makes it challenging to achieve participation in decision-making (20), especially within forensic psychiatric services due to their dual mission (52, 53). On the one hand, there is a caregiving role guided by the ethics of care, while on the other, there is a custodial role aimed at protecting the public from potentially dangerous behaviors. Staff members describe the balance between care and security as delicate in these settings (52, 54). Consistent with previous research (54, 55), participants in this study expressed a desire to participate in meetings about their care and to have greater influence over their treatment. However, they had limited opportunities to express their opinions, be taken seriously and influence their care and daily lives. It could be argued that listening to patients and providing greater opportunities for participation could be achieved without compromising safety (52, 54). Some researchers have highlighted the benefits of implementing shared decision-making within forensic psychiatric services (56), while others have emphasized the importance of building a strong therapeutic relationship and promoting a “realistic” level of patient participation (52). These perspectives reflect complementary aspects of the broader effort to enhance patient involvement in forensic psychiatric services. However, despite the ambitions expressed by staff, there still appears to be a lack of structural support enabling patients to participate in a meaningful way (21, 55, 57). The Safewards model was viewed by staff at the clinic as a tool to enhance patient participation, but it was not adopted by doctors or other professionals at the organizational level which limited its potential impact (58).

### 4.2 Reciprocal relationships

Creating an atmosphere of openness and support in forensic psychiatric services enables patients to feel safe and respected, which in turn can promote their well-being and rehabilitation as persons (59). Therefore, it is not surprising that patients have described connectedness to others as important (60). In our study, staff members were mostly described as available, positive and caring. However, some were perceived as stressed and unempathetic, hence when they were on duty, patients often remained in their rooms, the only space in which they felt safe. The participants were impressed by the ability of certain staff members to communicate with distressed patients. If staff members treat patients as persons worthy of respect and de-escalate conflicts, it increases the likelihood that patients will be open to learning how to control their emotions and actions. This in turn can provide opportunities for the patient to develop into a co-creator of a positive psychological climate on the ward (61). The study participants did not describe any co-creation processes or experiences of having their knowledge and personal insights valued or actively sought by staff. While meetings with staff were generally described as humane and compassionate, the organizational delivery of treatment and care was not perceived as person-centered or participatory. It was instead experienced as routine-driven and unresponsive to patients’ needs and wishes, thereby limiting opportunities for genuine social inclusion within the care environment (11).

### 4.3 Social justice

When it comes to social justice, it seems that staff in the present study were communicative. Furthermore, the participants did not report serious incidents of poor staff behavior, such as abuse of patients, harsh words or negative views of psychiatric patients, which frequently emerged in our earlier studies (21, 62). This may reflect the staff’s increased awareness and deliberate use of language promoted through the Safewards model (58). However, participants reported the same lack of activities and forums for meetings as in previous studies. Activities could serve as a means to promote citizenship and recovery, thereby enhancing social justice (31). The National Preventive Mechanism, which monitors Sweden’s compliance with the CAT, has also noted this problem and criticized a forensic psychiatric clinic for failing to adequately provide patients with opportunities for occupational therapy, daily outdoor access and other meaningful activities (No. O-4-2023) (6). A recurring issue in our study was the participants’ perception of unjustified confinement. This is linked to Sweden’s abolition of the insanity defense in criminal law (4). Historically, individuals who committed serious crimes could be discharged after a short forensic psychiatric care duration when no longer deemed to be suffering from a severe mental illness, a practice widely viewed as unjust by the general public. In response, legal amendments introduced mandatory special discharge reviews, effectively extending the duration of care for individuals convicted of serious offenses. However, at the same time, no equivalent measures were taken to prevent disproportionately long confinement for individuals convicted of minor offenses (4). Many patients remain in forensic psychiatric services after completing treatment, as the court deems the risk of recidivism to be high in cases where the municipality has not arranged appropriate housing and support for the individual, leading to considerable frustration. The National Preventive Mechanism has also criticized this practice (No. O-1-2021) (6), highlighting it as a violation of patients’ human rights, as it deprives them of their liberty without a justified need for psychiatric treatment. These social justice issues cannot be solely addressed through the implementation of Safewards. There is a need for Swedish forensic psychiatric services to be more accountable, transparent and place greater emphasis on patients’ human rights, in the same way as the Comprehensive Framework to Advance Equity, Diversity, and Inclusion in a Forensic Service in Ontario, Canada (63).

### 4.4 The safewards model and social inclusion

Safewards’ potential for enhancing patients’ social inclusion has yet to be fully achieved in the clinic in which the study was conducted. In another study conducted at the same clinic, staff at the clinic reported actively working to become more care-oriented (58), something that our participants had noted. However, there was also resistance to the implementation of Safewards on the part of some staff members, which is supported by other studies (64, 65). The clinic environment was perceived as dull, and participants were disappointed at not being involved in the development of *Clear Mutual Expectations*. A key area for improvement lies in incorporating greater co-creation into the implementation process. *Mutual Help Meetings* could be used to promote patient engagement, but none of the participants reported positive experiences of these meetings. To achieve more meaningful outcomes, it may be necessary to implement *Mutual Help Meetings* in a way that aligns more closely with the core principles of the intervention. These meetings, together with *Everydayness*, an intervention proposed in Safewards Secure (40), could serve as a platform for reshaping daily life on the ward, fostering a sense of community and collaboration. Involving patients in the co-creation of Safewards interventions and social activities would also be in line with human rights values, like social justice and non-discrimination (14).

### 4.5 Is social inclusion possible in forensic psychiatric services?

A literature review on recovery in forensic psychiatric services suggests that social inclusion and normality are primarily linked to integration into society as a citizen (66), whereas other perspectives emphasize that institutional settings themselves can be adapted to support social inclusion (67). Given our definition, forensic psychiatric patients are also citizens with rights, making social inclusion a relevant goal within institutions. However, as this study and existing literature indicate, inclusion remains insufficient. The main reason is that the shift toward a more compassionate approach by staff does not appear to have been accompanied by organizational changes supporting participation and person-centeredness. Instead, existing structures tend to reinforce a routine-based model of care. Organizational barriers to person-centered care have been described as deeply rooted in a long-standing biomedical and positivist paradigm. This paradigm emphasizes professional rather than personal goals, relies on objective measures and evaluations, and positions patients as passive recipients of care. Successful efforts to overcome these barriers have included sustained leadership commitment and multidisciplinary teamwork aimed at changing routinized practices and communication strategies (68).

### 4.6 Strengths and limitations

This study offers new perspectives on Safewards, due to employing a value-based analysis, which has not previously been used. One of the study’s strengths is the interviewers were experts by experience. Another is that the analysis was carried out by researchers with diverse research backgrounds and varying experiences of forensic psychiatry as staff, patients, researchers and students. Combined with reflective practice, this contributed to a robust interpretation of the interview data.

However, the study has certain limitations. At the time of data collection, each ward had adopted a different subset of Safewards interventions, and several patients had been transferred between wards, resulting in overlapping experiences. Given this mobility and the small sample size, stratifying the analysis by ward was not feasible. Therefore, the analysis was conducted at the clinic level. The sample consisted of eight male patients from a single clinic which is a relatively small number and makes the findings context specific. It is also a limitation that we have no experiences from female patients, since none of the three female patients at the clinic met our inclusion criteria. A common challenge in conducting interviews within forensic psychiatric services is the limited control over participant selection. For this reason, we chose from the outset to invite all patients who were eligible and available to participate. Another problem was that we would have preferred a richer dataset, as some patients occasionally had difficulty expressing themselves. We aimed to obtain longer, coherent narratives, but this was often impossible. Therefore, we also include quotations that illustrate the communication between the participants and the interviewers to provide insight into the nature of their interactions. A further limitation concerns the exclusion of non-Swedish-speaking patients and others with limited language proficiency. This decision was made for practical reasons and because of the abstract nature of the interview questions. Communication and language barriers remain a significant human-rights and equity concern (9). Future research should explore multilingual or observational approaches to better include these patient groups. Nevertheless, we believe that these patient voices can contribute to a deeper understanding of what social inclusion may mean for patients in forensic psychiatric care. However, there is also a need for research exploring the processes of discharge and community reintegration, and how it may influence the possibility for social inclusion of former patients in society.

## 5 Conclusions

This study demonstrates that the social inclusion model has managed to capture several important aspects of human rights. The implementation of Safewards seems to have the potential to enhance human rights and, consequently, promote socially sustainable forensic psychiatric services. Above all, it appeared that Safewards promoted reciprocal relationships and a more compassionate approach by staff but failed to change the organization in terms of more participatory and person-centered care. However, certain essential aspects of social justice—such as prolonged care duration and the right to a meaningful everyday life—cannot be fully achieved without legislative and policy reforms. Ensuring social sustainability in forensic psychiatric services requires a fundamental cultural shift, with an explicit emphasis on human rights alongside the implementation of Safewards. This study focuses on social inclusion in the institutional environment.

## Data Availability

Data are not available since this could compromise the individual privacy of participants. The data are stored at the University Health Care Research Center, Region Örebro County, and may be available on request by other researchers, without undue reservations. Further enquires can be directed to the corresponding author.
