# Supplementary material for: Between rights and reality: patient perceptions of social inclusion in a forensic psychiatric clinic implementing safewards
Source: Front Psychiatry. 2025 Nov 19;16:1688564. doi: 10.3389/fpsyt.2025.1688564 (PMC12673394; doi:10.3389/fpsyt.2025.1688564)
Supplement: Supplementary file 1 [file Supplementaryfile1.docx]

# Guide for Individual Interviews with Patients

## Introduction

1. The interviewers introduce themselves, their role in the research project, their profession, and their own experience with psychiatric care.
2. Briefly explain the background and purpose of the study and present the topic for the interview.
3. Assure confidentiality and inform participants that they can withdraw at any time.
4. Explain how the material will be handled – recording, transcription, reporting, storage, and confidentiality.
5. Allow participants to ask questions.
6. Ask participants to sign the consent form.
7. Ask participants if they wish to use their real name or a pseudonym and ask about their age.
8. Ask if participants would like feedback on the study’s results.

### Study Aim and Questions

This ward has been working to implement Safewards for some time. Safewards consists of various components. The aim of this study is to explore how you experience being cared for on this ward and how you perceive the impact of Safewards on the ward environment.

## Interview Questions

1. What is it like to be a patient on this ward?
2. Differences compared to other wards?

### Interview Topics for the Interviewer to Use:

#### Social Inclusion and Participation

- Can you reflect on what characterises this ward and its culture or environment in terms of:
  1. Sense of safety and security/insecurity and feeling unsafe on the ward – what makes you feel safe?
  2. Feelings of care, comfort, and hope from staff and fellow patients vs. indifference, detachment, or coldness.
  3. Feeling seen as a person rather than just a patient, participation in decision-making – participation in implementation, care, and daily life.
  4. Supportive vs. obstructive experiences.
  5. Compassion and a sense of unity vs. objectification, distance, an ‘us-and-them’ mentality, or harshness.
  6. Group activities and participation in them.
  7. Positive ward atmosphere vs. negative ward atmosphere.
  8. Equality in human dignity, respect, treatment, and interactions vs. unequal in human dignity, respect, treatment, and interactions.
  9. A shared perspective on patients among staff vs. differing views. Individualised care, being seen as a unique person.
  10. Emphasising strengths and positives vs. focusing on negatives and breaking people down.
  11. Feeling included, remembered, and involved vs. exclusion and feeling like an outsider. Staff availability and presence – support in managing difficult emotions.

#### Experiences and Perspectives on Safewards

A paper is handed out with the name, and a summary, of each Safewards intervention used on the ward, for summary and questions about implemented interventions, see pages 3-6.

#### Safewards’ Contribution to a Safe Ward Environment

One goal of Safewards is to contribute to a safe ward environment. Based on this goal, what are your reflections on:

1. What does safety mean to you?
2. How do you experience it? Safewards’ contribution to:
   - overall safety on the ward?
   - safety among patients?
   - safety between patients and staff?

#### Summary and Conclusion

1. If possible, provide a summary or key points from the interview and ask participants if they agree with the summary or if they have anything to add.
2. Thank the participants and ask if they have any further questions about the study.
3. Remind participants how they can receive feedback on the study’s findings if they wish.

# Interview guide. The Safewards Interventions

*Clear Mutual Expectations*

There is a poster on the ward outlining the *Clear Mutual Expectations* on the unit (instead of rules for patients).

1. Are you aware of the *Clear Mutual Expectations*?
2. If yes, what are your thoughts about them?
3. Possible follow-up questions:
   1. Have the *Clear Mutual Expectations* been helpful or positive for you in any way?
   2. Have the *Clear Mutual Expectations* been negative for you in any way?
   3. Do you know how *Clear Mutual Expectations* have affected other patients? If so, how?
   4. What could the staff do more to improve this intervention?

*Know Each Other*

There is a binder on the ward with presentations of the staff (and patients?) to facilitate everyday conversations.

1. Are you aware of this binder?
2. If yes, were you offered to write something in it? (According to the instructions, patients are also encouraged to introduce themselves.)
3. What are your thoughts about the *Know Each Other* intervention?
4. Possible follow-up questions:
   1. Has the *Know Each Other* intervention been helpful or positive for you in any way?
   2. Has the intervention been negative for you in any way?
   3. Do you know how this intervention has affected other patients?
   4. What could the staff do more to improve this intervention?

*Discharge Messages*

There is a tree on the ward, which is meant to represent *Discharge Greetings*. The purpose is to inspire hope.

1. Are you aware of the tree?
2. If yes, what are your thoughts about it?
3. Possible follow-up questions:
   1. Have you found the tree helpful or encouraging in any way?
   2. Has the tree been negative for you in any way?
   3. Do you know if and how the tree has affected other patients?
   4. What could the staff do more to improve this intervention?

*Mutual Help Meeting*

The staff organizes a *Mutual Help Meeting* on the ward so that you can inform each other about the current situation and support one another.

1. Are you aware of these meetings on the ward?
2. If yes, have you participated in any of these meetings?
3. What are your thoughts about the *Mutual Help Meeting*?
4. Possible follow-up questions:
   1. Have you found these meetings helpful or beneficial in any way?
   2. Has participating in any of these meetings been negative for you in any way?
   3. Do you know if and how other patients have been affected by these meetings?
   4. What could the staff do more to improve this intervention?

*Soft Words*

Staff on the ward are expected to communicate kindly when saying no, asking patients to do something, or asking them to stop doing something that may disturb others.

1. Have you noticed how the staff communicate when they say no, ask you or other patients to do something, or to stop a certain behaviour?
2. If yes, what happened?
3. Possible follow-up questions:
   1. Can you tell more about what happened before/during/after?
   2. Do you have more examples of how the staff use this approach?
   3. Have you noticed any situation where the intervention didn’t work?
   4. Have you noticed any situation where the staff didn’t use *Soft Words* even though it would have been appropriate?
   5. What could the staff do more to improve this intervention?

*Bad News Mitigation*

When a patient receives difficult or negative news, the staff on the ward are expected to deliver it in a calm and supportive manner.

1. Have you or another patient you know of received negative news during your time on the ward?
2. If yes, what happened?
3. Possible follow-up questions:
   1. How do you feel the staff responded to your or the other patient’s need for support?
   2. Can you tell more about what happened before/during/after?
   3. Do you have more examples of how the staff use this intervention?
   4. Have you noticed any situation where the staff did not manage to deliver negative news in a gentle way?
   5. What could the staff do more to improve this intervention?

*Calm Down Methods*

If a patient is struggling with their emotions, the staff are expected to offer *Calm Down Methods*, such as stress balls, herbal tea, etc.

Have you noticed staff helping you or another patient with *Calm Down Methods*?

1. If yes, what happened then?
2. Possible follow-up questions:
   1. How do you feel the staff responded to your or the other patient’s need for support?
   2. Can you tell more about what happened before/during/after?
   3. Do you have more examples of how the staff use *Calm Down Methods*?
   4. Have you noticed any situation where the intervention wasn’t used or didn’t work?
   5. What could the staff do more to improve this intervention?

*Talk Down*

Staff on the ward are expected to respond to upset, anxious, or aggressive patients in a calm and understanding manner in order to de-escalate the situation.

1. Do you have any experience of either yourself or another patient being very upset, anxious, or aggressive?
2. If yes, what happened then?
3. Possible follow-up questions:
   1. How do you feel the staff responded to you or the other patient in that situation?
   2. Can you tell more about what happened before/during/after?
   3. Do you have more examples of how the staff use the *Talk Down* intervention?
   4. Have you noticed any situation where this intervention didn’t work or wasn’t applied as intended?
   5. What could the staff do more to improve this intervention?

*Reassurance*

If something happens on the ward that could be perceived as frightening by patients – for example, when another patient is feeling very unwell and the situation becomes tense – the staff are expected to talk with patients about it and provide information to help everyone feel safe.

1. Have you experienced an incident where you or another patient felt frightened or worried?
2. If yes, how did the staff respond? What happened?
3. Possible follow-up questions:
   1. How do you feel the staff responded to your or other patients’ need for support?
   2. Can you tell more about what happened before/during/after?
   3. Do you have more examples of how the staff use this intervention?
   4. Have you noticed any situation where the intervention wasn’t used or didn’t work even though something happened that made patients feel uneasy?
   5. What could the staff do more to improve this intervention?
